# Hawkmoths can smell with grooming organs on their legs

**DOI:** 10.1007/s00359-025-01769-y

**Published:** 2025-10-25

**Authors:** Ahmed Reda Ismaieel, Regina Stieber, Bill S. Hansson, Sonja Bisch-Knaden

**Affiliations:** 1 Department of Neuroethology, Max Planck Institute for Chemical Ecology, Hans-Knoell- Straße 8, 07745 Jena, Germany; 2 Entomology Department, Faculty of Science, Ain Shams University, Abbassia, 11566 Cairo Egypt

**Keywords:** Moth, Epiphysis, Olfactory sensillum, Odorant receptor, Ionotropic receptor, Gustatory receptor, Electrophysiological recordings, Oviposition

## Abstract

The antennae are the primary olfactory organs of insects, though other appendages, such as mouthparts and the female ovipositor, can also detect odors. A prerequisite for the olfactory function of an appendage is the presence of sensilla with porous walls and the expression of chemosensory receptors by sensory neurons housed in these sensilla. In the tobacco hawkmoth, *Manduca sexta*, we demonstrate that the epiphysis, a small process on the tibia of the forelegs that is used to clean the antennae, is an olfactory organ. The epiphysis carries approximately 150 sensilla with wall pores, suggesting an olfactory function. Additionally, the epiphysis expresses a variety of chemosensory receptor genes. We identified the expression of *ORCo*, the obligate co-receptor of odorant receptors (*ORs*), as well as 54 tuning *ORs*. Moreover, the epiphysis expresses 22 ionotropic receptors (*IRs*), including the co-receptors *IR8a*, *IR25a*, and *IR76b*, and 33 gustatory receptors (*GRs*). Several of these *IRs* and *GRs* had not previously been found in the antennae or other appendages. Electrophysiological recordings from isolated epiphyses revealed responses to odorants from several chemical classes, host plant leaves, and the female pheromone gland. The strongest responses were elicited by acids and the amine pyrrolidine. Epiphysectomy did not affect courtship or foraging behavior; however, epiphysectomized females were less likely to reach a host plant than controls. Our study reveals the epiphysis of *M. sexta* to be a previously unknown olfactory appendage with a broad and partly unique chemosensory repertoire. Because the epiphysis is a constitutive feature of lepidopteran insects, its olfactory function may be present in most moths and butterflies.

**Supplementary Information:**

The online version contains supplementary material available at 10.1007/s00359-025-01769-y.

## Introduction

One of the common morphological features in the ground plan of moths and butterflies is the epiphysis, a short process on the inner surface of the foreleg tibia of both females and males (Kristensen et al. 2007). The epiphysis is densely covered with comb-like cuticular structures, and the size of this tissue usually correlates with the size of the antenna (Philpott 1924). These morphological findings, together with anecdotal observations, led to the idea that the epiphysis is an antenna-grooming organ. Experiments with fluorescent powder or pollen grains applied to the antenna have shown that moths and butterflies do use their epiphyses to clean the antenna, as the particles were consistently transferred to the epiphyses (Callahan and Carlysle 1971; Odell et al. 1982; Robbins 1989). An additional function of the epiphysis has been suggested in a study of the moth *Lymantria dispar* (Odell et al. 1982). Males of this species that had their epiphyses surgically removed had the same mating success rate as control males. However, the epiphysectomized males took longer to mate, suggesting that the epiphysis might play a role in precopulatory behaviors. As courtship in moths largely relies on olfactory communication, we asked whether the epiphysis might have an olfactory function beyond being a grooming organ.

To answer this question, we studied the epiphysis of the hawkmoth *Manduca sexta*, a moth with a well-characterized olfactory system with respect to the antenna, the main olfactory organ of insects (Kalinova et al. 2001; Sanes and Hildebrand 1976; Shields and Hildebrand 2001), as well as other appendages with olfactory function, such as the labial palps (Kent et al. 1986), the proboscis (Haverkamp et al. 2016), and the ovipositor (Klinner et al. 2016). In addition, members of the three major families of chemosensory receptor genes — odorant receptors (*ORs*), ionotropic receptors (*IRs*), and gustatory receptors (*GRs*) — have been annotated (Koenig et al. 2015), and the expression of at least some of these receptors has been detected in different appendages of *M. sexta*, including the forelegs (Tom et al. 2022).

Precopulatory behavior in *M. sexta* involves the male, after approaching a female, climbing onto the back of her thorax and touching her with his foreleg tarsi before the pair assumes the final mating position with heads facing opposite directions (Lingren et al. 1977). During the climbing phase, the male’s epiphyses are in near proximity to the female, and close-range detection and evaluation of the pheromone blend or other volatiles emitted by the female is theoretically possible. In addition, the epiphysis might be involved in other behavioral contexts such as oviposition. In female moths and butterflies, contact chemosensilla on the foreleg tarsi are used to evaluate the identity and quality of an oviposition substrate (Ozaki et al. 2011; Takai et al. 2018; Tsuchihara et al. 2022). Because the epiphysis is usually close to, but not in contact with, the leaf surface or other substrates, it may function as an olfactory organ rather than a contact chemosensory organ when involved in oviposition behavior.

In the present study, we explored the potential olfactory capacity of the epiphysis of *M. sexta* using morphological, electrophysiological, behavioral, and gene expression analyses.

## Materials and methods

### Insect rearing

*M. sexta* was reared in our laboratory on an artificial diet (Grosse-Wilde et al. 2011). Larvae were kept in a climate chamber with a 14-h light/10-h dark cycle at 26 °C during the light cycle and 24 °C during the dark cycle and a relative humidity of 60%. After pupation, male and female pupae were kept in separate climate chambers, with a 16-h light/8-h dark cycle at 25 °C and relative humidity of 60% during the light cycle and 70% during the dark cycle. Emerging adults were collected daily and individually housed in brown paper bags (17 cm × 26 cm) in the pupal chambers.

### Scanning electron microscopy (SEM)

The epiphysis was cut from the tibia of the foreleg with a pair of curved microscissors, and the scales covering the epiphysis were removed with a toothpick. The samples were dehydrated by repeated washing in 70% ethanol, then placed in 1.5 ml Eppendorf tubes on tissue and air-dried for at least 24 hs. The epiphyses were mounted on a holder covered with adhesive tape and sputter-coated with gold before examination with a scanning electron microscope (LEO 1450 VP, Zeiss, Germany).

### Test for antennal grooming

To evaluate whether the epiphysis of *M. sexta* plays a role in antenna cleaning, we applied a small amount of fluorescent powder (DayGlo, USA) with a toothpick to the epiphysis of the right foreleg of a 3-day-old male moth, leaving the left leg untreated as a control. The moth was then placed in a mesh cage (40 cm x 40 cm x 55 cm) in a climate chamber with a 16-h light/8-h dark cycle at 25 °C and relative humidity of 60% during the light cycle and 70% during the dark cycle. After 24 h, the moth was placed in a freezer for 2 days, and then both antennae and epiphyses were examined under a microscope (Axio Zoom, Zeiss, Germany).

### Electrophysiology

The electroepiphysisogram (EEG) was developed as an adaptation of the electroantennogram (EAG). For this recording technique, an epiphysis was dissected from the foreleg and attached to two steel electrodes (‘recording fork’) with conductive gel (Spectra 360 electrode gel, Parker Laboratories) after cutting off a small portion of the epiphysis tip. In addition, we performed single sensillum recordings (SSR) from individual sensilla on the epiphysis. For this purpose, the epiphysis was cut together with a small part of the tibia to provide an area for the grounding electrode. The tungsten recording electrode was inserted into the base of a sensillum.

A constant flow (0.5 l/min) of charcoal-filtered and humidified air was delivered through an aluminum tube (length: 11 cm), with the outlet positioned 1–2 cm from the epiphysis. Ten µl of the odor stimulus was pipetted onto a filter paper disk (diameter: 1.2 cm) placed inside a glass Pasteur pipette. The tip of this Pasteur pipette was inserted into a small hole in the aluminum tube. For odor stimulation, an airstream (0.4 l/min) was delivered through the Pasteur pipette into the continuous airstream for 200 ms (CS-55 Stimulus Controller, Syntech, Germany). The signals were digitally converted (IDAC-4 USB, Syntech, Germany), visualized, and recorded on a PC using the software Autospike (Syntech, Germany).

To analyze the EEG and EAG data, the maximum deviation from baseline, i.e., the amplitude, was determined for each experiment. At the beginning and end of the sequence of odor stimuli tested with an epiphysis or antenna, a control stimulus using solvent was done. The average amplitude elicited by these two control stimulations was calculated and subtracted from the amplitude elicited by each odor stimulus. This provided the solvent-subtracted EEG or EAG response.

### Odor stimuli

 Twenty-five synthetic odorants (Table S1) were diluted in hexane to a concentration of 10^−2^.

To prepare a female pheromone gland extract, we dissected the glands of ten 3-day-old virgin females at five hours into the scotophase, when females are most attractive to males (Allen and Hodge 1955). The glands were immersed in 500 µl hexane and placed on a shaker for 1 h. Ten µl of the supernatant was used per stimulation, corresponding to 0.2 female gland equivalents (FGE), a concentration within the range of behavioral attractiveness (0.002 to 2 FGE) (Doolittle et al. 1991).

To collect plant headspace, a non-flowering *D. wrightii* plant (potted) or a single flower from a potted *D. wrightii* plant was enclosed in a polyethylene terephthalate bag (Toppits, Germany). Charcoal-filtered air was pumped into the bag through a silicone tube connected to a custom-made pump. The odor-enriched air exited the bag through a second silicone tube that passed through a volatile collection trap (Porapak-Q 25 mg, https://www.volatilecollectiontrap.com). Volatile collection was done in a climate chamber with a 14-h light/10-h dark cycle at 25 °C (day) and 22 °C (night) and relative humidity of 57% during the light cycle and 65% during the dark cycle. After 24 h, the traps were removed and eluted with 400 µl hexane.

### Tissue collection and RNA extraction

We studied the expression of chemosensory receptor genes in the epiphyses of male and female moths, both virgin and mated, on day 3 after eclosion. Mating took place on day 2 after eclosion. For RNA extraction, three pairs of epiphyses, i.e., epiphyses from three animals, were pooled per sample, and three samples were prepared for each experimental group (virgin males, mated males, virgin females, and mated females). Tissues were cut from the forelegs and immediately pestled in liquid nitrogen using a mortar containing 1.5 ml of TRI Reagent (Sigma Aldrich, USA). The resulting mixture was transferred to a 2 ml Eppendorf tube. After this step, we followed the manufacturer’s protocol (Direct-zol RNA Miniprep Kits). The total RNA concentration per sample was 30–40 ng/µl. The number of *ORs*, *IRs*, and *GRs* detected in the epiphysis is higher than in a recent study that examined the expression of chemosensory receptor genes in the foreleg of *M. sexta* using the same technique (Tom et al. 2022), probably because the former study extracted RNA from the entire leg, thereby diluting the mRNA copies of these receptors. However, all chemosensory receptor genes (except *MsexOR8*) previously found in the entire foreleg were also expressed in the epiphysis.

### NanoString gene expression assay

We used the nCounter XT CodeSet gene expression assay (NanoString Technologies, Inc., USA). The custom CodeSet (Zhang et al. 2022) contained 268 probes targeting 71 ORs, 29 IRs, 49 GRs, 47 odorant-binding proteins, 5 pickpocket, 3 sensory neuron membrane proteins, and 62 candidate reference gene transcripts. Some receptor sequences had high homology with duplicates, preventing the design of unique probes, and therefore had to be excluded (Tom et al. 2022). We followed the standard protocol described in the nCounter XT Gene Expression Assay User Manual (MAN-10023-11, page 16). Since *MsexABPx*, *MsexOBP1*, *MsexOBP5*, and *MsexOBP6* had very high expression levels, an attenuation mix (Eurofins Genomics, Germany) was used to suppress these counts.

For the hybridization step, we prepared a master mix of 42 µl Reporter CodeSet, 28 µl Reporter-Plus reagent, and 70 µl nCounter SPRINT hybridization buffer. Each hybridization reaction combined 10 µl master mix, 5 µl total RNA (30–40 ng/µl RNA), 1 µl attenuation mix, and 3 µl of a mixture consisting of Capture ProbeSet and Capture-Plus reagent. Hybridization was done at 65 °C for 22 h, after which 16 µl Merck water was added to the sample. The total volume was loaded onto the nCounter SPRINT Cartridge and processed on the nCounter SPRINT Profiler. Raw data was processed using nSolver4.0. Quality control of the mRNA data was done using default parameters for the nCounter SPRINT Profiler according to the NanoString Gene Expression Data Analysis Guidelines (MAN-C0011-04). The parameters were Imaging QC: 75; Binding Density QC: 0.1–1.8; Positive Control Linearity QC: 0.95; Positive Control Limit of Detection QC: 2 standard deviations. Two normalization steps were then performed, first using the geometric mean counts of six external positive control probes and second using the geometric mean counts of at least three endogenous reference genes selected based on their coefficient of variation (CV). The endogenous reference genes used for the second normalization step were msex02_01637RB, msex02_11794RA and msex02_13396RA with CV < 40%. After these two normalization steps, the minimum normalized value for each sample was defined as background, and any chemosensory receptor gene with values above this background in at least two of the three samples was considered to be expressed in that experimental group.

### Reverse-transcriptase PCR

To clarify the expression of the OR co-receptor *ORCo* and the IR co-receptors *IR8a*, *IR25a*, and *IR76b* in the epiphysis, RNA extracted from female and male epiphyses and from male antennae (positive control) was used to synthesize cDNA with the Superscript III Reverse Transcriptase Kit (Thermo Fisher Scientific, Germany). To amplify the genes, PCR was performed with Phusion™ High-Fidelity DNA Polymerase (New England Biolabs, Germany) according to the manufacturer’s protocol and the primers in the table below at an annealing temperature of 60 °C. The size of the PCR products was visualized and analyzed with gel electrophoresis.

**Table:** 

Gene	Forward primer	Reverse primer
*MsexORCo*	ATGATGGCCAAAGTGAAAACACAGG	CTATTTCAGCTGCACCAACACCATG
*MsexIR8a*	AAGAGCAGTGAAAGAGAAGTTAGTGCGC	TCCACACCCTGTAAAGTGTGTCTTCTG
*MsexIR25a*	ATGTTATCAGCGAAAAAGACTCCTCACGTC	TCAAAATTTAGGTTTCAAATTAGATAAACCTAAATTTC
*MsexIR76b*	ATGGCCGGGATCGAGCTCATTATATC	TTATCGATACAGAAAAGCAGAAGGCGCTC

### Mating experiments

To test the effect of the epiphysis on mating success, both epiphyses of one sex were removed during the inactive (light) phase on the day of eclosion. The control animals were handled in the same way and for the same amount of time, but without removing the epiphyses (“mock surgery”). On the third day after eclosion, individuals of the epiphysectomized moths were then allowed to mate with a control animal of the opposite sex in a Plexiglas mesh cage (30 cm x 30 cm x 30 cm) during the active (dark) phase. Females were placed in the cages at the beginning of the dark phase, and males were added 4 h later. Cages were then observed for 60 min, and the time of onset of copulation was noted.

### Wind tunnel experiments

To test the effect of the epiphysis on feeding and oviposition behavior, we removed the moths’ epiphyses on the day of eclosion during the light phase. The control animals were handled in the same way and for the same amount of time, but without removing the epiphyses (“mock surgery”). Experiments were performed on the third day after eclosion, either with virgin moths (males and females) or after mating on the second day (females). We conducted the experiments during the active phase of the moths in a Plexiglas wind tunnel (250 cm long x 90 cm wide x 90 cm high) at 25 °C, 70% relative humidity, and a wind speed of 44 cm/s. Individual moths were transferred to a plastic mesh cylinder (15 cm x 14 cm) and placed in an acclimation chamber with conditions similar to those in the wind tunnel for at least 1 h prior to the start of the experiment. At the downwind end of the wind tunnel, a moth was placed on a 40 cm platform, while at the upwind end, either a single *D. wrightii* flower attached to a 50 cm pole (feeding experiments), or a pot with a non-flowering three-leafed *D. wrightii* plant (oviposition experiments) was placed. Moths that were unable to fly or did not initiate wing beats within 2 min were excluded. Flying moths were observed for 3 min and filmed using a Sony Handycam DCR-SR35 in night shot mode. We counted the number of moths that touched the flower with the tip of their proboscis (feeding experiments) and the number of moths that touched a leaf with their tarsi (oviposition experiments), and calculated the total duration of these contacts per animal. In oviposition experiments, we counted the number of eggs laid on the leaves.

### Statistics and figures preparation

Sample size and statistical tests are described in both the text and figure legends. Statistical analyses were done with GraphPad InStat (version 3.10, GraphPad Software, San Diego, CA, https://www.graphpad.com). Figures were generated using PAST (version 3.26, http://folk.uio.no/ohammer/past/), RStudio, GraphPad Prism9 (https://www.graphpad.com), and edited using Adobe Illustrator CS5.

## Results

### Morphology of the epiphysis in *M. sexta*

**Fig. 1:**
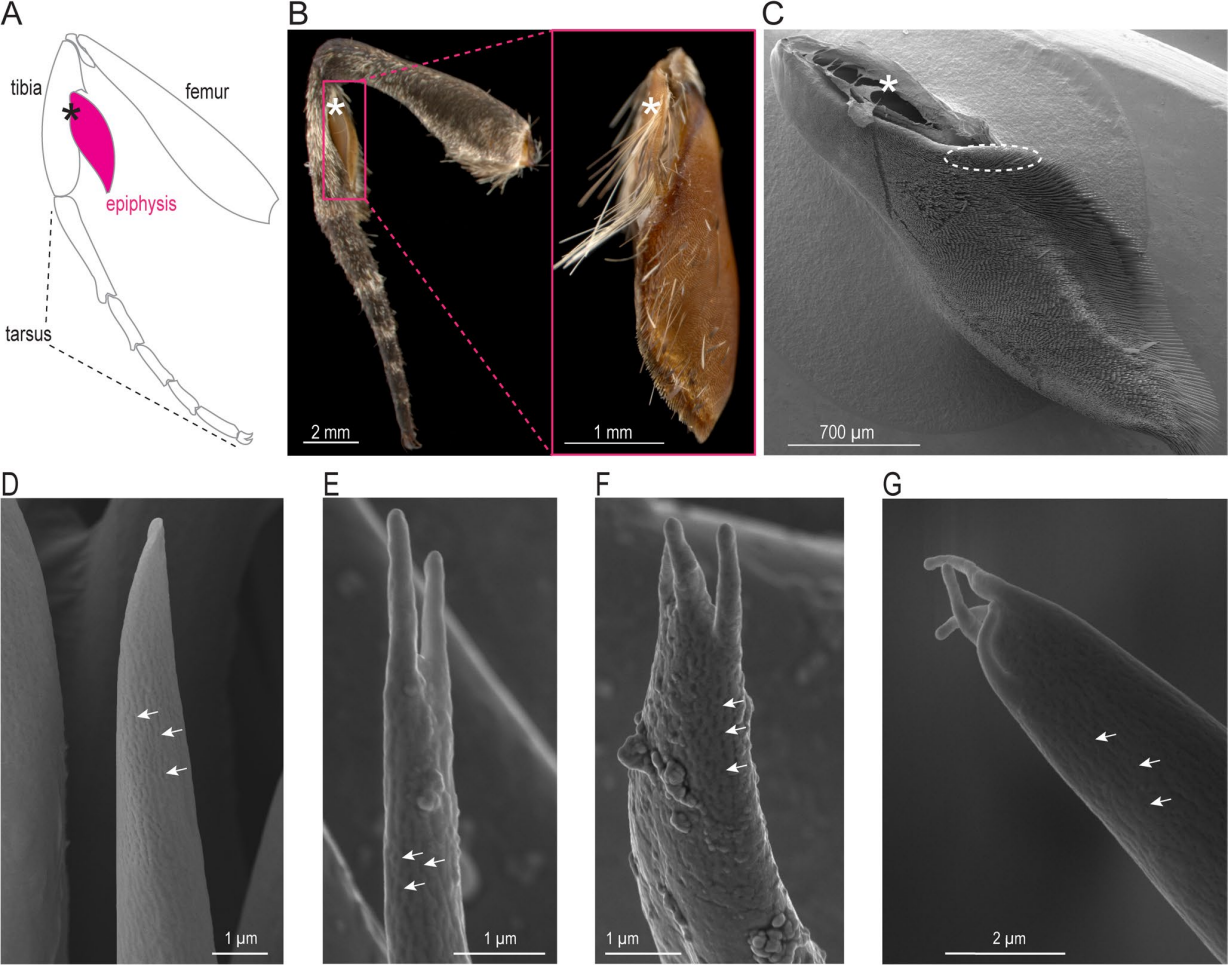
Position of the epiphysis on the foreleg of *M. sexta* and putative olfactory sensillum types present on the epiphysis. **A** Schematic of the foreleg (modified from Madden 1944). *Asterisk*, insertion site of epiphysis on tibia. **B** *Left*, light microscopic image of a male foreleg. *Pink box*, epiphysis; *right*, close-up of epiphysis; *asterisk*, insertion site of epiphysis on tibia. **C** Scanning electron microscopy image of a male epiphysis. *White dashed outline*, region with putative olfactory sensilla; *asterisk*, insertion site of epiphysis on tibia. **D–G** Sensilla within the white dashed outline in C. Sensilla in E-G have 2–4 finger-like terminal structures; *arrows*, wall pores.

The epiphysis is an elongated, shoe-like tissue with its proximal end attached to the foreleg tibia of both male and female moths (Fig. 1A, B). As in other moth species (Ancajima et al. 2025; Odell et al. 1982; Philpott 1924), the epiphysis of female *M. sexta* is smaller than that of males (Table 1), and its surface facing the tibia is covered by rows of comb-like cuticular structures (acanthae, (Richards and Richards 1979). To confirm that *M. sexta*, like *M. quinquemaculata* and other moths (Callahan and Carlysle 1971; Odell et al. 1982) cleans its antenna with the epiphysis, we applied a small amount of fluorescent powder to the epiphysis of one foreleg, while the epiphysis on the other foreleg was untreated. After 24 h, we found fluorescent powder only on the antenna on the same side as the powdered epiphysis, but not on the other side (Fig. S1), suggesting that the moth had pulled its antenna between the epiphysis and the tibia and transferred the powder from the treated side to the antenna while attempting to clean it.

However, examination of the cuticular structures covering the epiphysis revealed a small region (Fig. 1C) with 40–80 μm long sensilla, many of which seemed to have perforated walls (Fig. 1D-G, arrows), a typical feature of sensilla with an olfactory function. Sensilla with a single apical pore, indicating a gustatory function, were not found within the array of sensilla with wall pores nor on the remaining surface of the epiphysis. The putative olfactory sensilla occurred in similar numbers of 150–156 in females and males (Table 1), and either resembled antennal basiconic sensilla (Fig. 1D) or had 2–4 terminal finger-like cuticular processes (Fig. 1E-G). The epiphyses on the tibia of the foreleg of *M. sexta* may therefore be part of the olfactory system of the moth.

**Table 1:** Morphometric characteristics of the epiphysis of female and male *M. sexta*

	Females	Males	t-test
Length of epiphysis [mm]*	2.9 ± 0.3 (*n* = 10)	3.4 ± 0.3 (*n* = 10)	*p* < 0.001
Width of epiphysis [mm]*	0.8 ± 0.1 (*n* = 10)	1.1 ± 0.2 (*n* = 10)	*p* < 0.001
# Putative olfactory sensilla*	156 ± 20 (*n* = 3)	150 ± 21 (*n* = 3)	*p* = 0.8

*Mean ± standard deviation (sample size)

### Is the epiphysis able to smell?

**Fig. 2:**
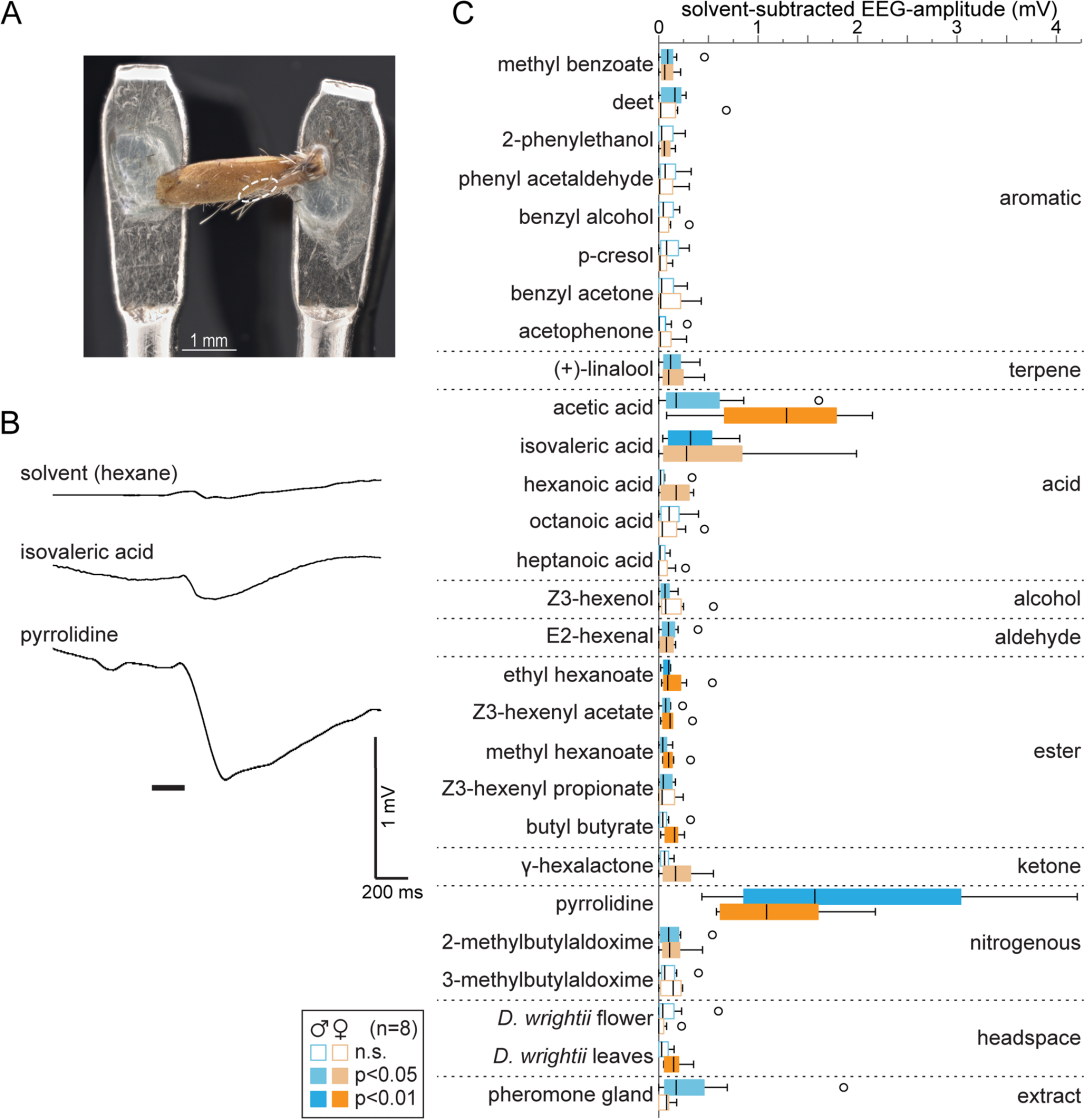
Electrophysiological recordings from the epiphysis (EEG). **A** Picture of a female epiphysis attached to the recording electrodes with conductive gel. *Dotted white line*, array with putative olfactory sensilla. **B** Representative EEG recordings showing responses of a female epiphysis to the solvent hexane and two odors; *black bar*, stimulus (200 ms). **C** EEG responses of male (blue) and female (orange) *M. sexta* epiphyses to 25 odorants from eight chemical classes (10 µl, diluted 1:100), headspace (10 µl) of *D. wrightii* (flower and leaves separately), and extract (10 µl) from female pheromone glands. *Boxplots*, median, interquartile range and range; *circles*, outliers; *filled boxes*, data different from zero (*p* < 0.05 (light blue/orange) or *p* < 0.01 (dark blue/orange), Wilcoxon signed rank test); results did not differ between the sexes (*p* > 0.05, Mann-Whitney U test); see Fig. S2 for a comparison with results from corresponding EAG recordings to the same stimulants; the Bonferroni-Holmes method was used to adjust the significance level for multiple comparisons

To test the ability of the epiphysis to detect odors, we developed a modified electro-antennogram (EAG) technique to record odor-evoked responses from isolated epiphyses (Fig. 2A). For this electro-epiphysisogram (EEG), we used 25 synthetic odorants (Table S1). Most of these odorants were chosen because they induced feeding and/or oviposition behavior in previous wind tunnel experiments with female *M. sexta* (Bisch-Knaden et al. 2018). Other odorants from different chemical classes were included to broaden the range of chemical stimuli. Additionally, the headspace of *Datura wrightii* flowers and leaves, as well as an extract from the female pheromone gland were used as complex, natural cues. *D. wrightii* is a valuable nectar source and larval host plant of *M. sexta*.

We found that 10 out of the 25 tested odorants elicited significant responses in the epiphysis of both male and female moths when compared to solvent controls. Another six odorants elicited responses in one sex (Fig. 2B, C). The active odorants belonged to each of the tested chemical classes. Pyrrolidine and two acids (acetic acid and isovaleric acid) were the most potent stimulants. Leaves of the host plant elicited a response in female epiphyses, and extract from the female pheromone gland activated male epiphyses. Because the antennae have more olfactory sensilla and express more olfactory receptors than the epiphyses, antennal responses are likely stronger. EAG recordings using the same stimulants confirmed this assumption. However, for certain odors, such as acetic acid and pyrrolidine, the responses were similar in both tissues (Fig. S2). Thus, our electrophysiological results suggest that functional olfactory sensory neurons are present in the epiphysis of *M. sexta*.

### Does the epiphysis express chemosensory receptors?

**Fig. 3:**
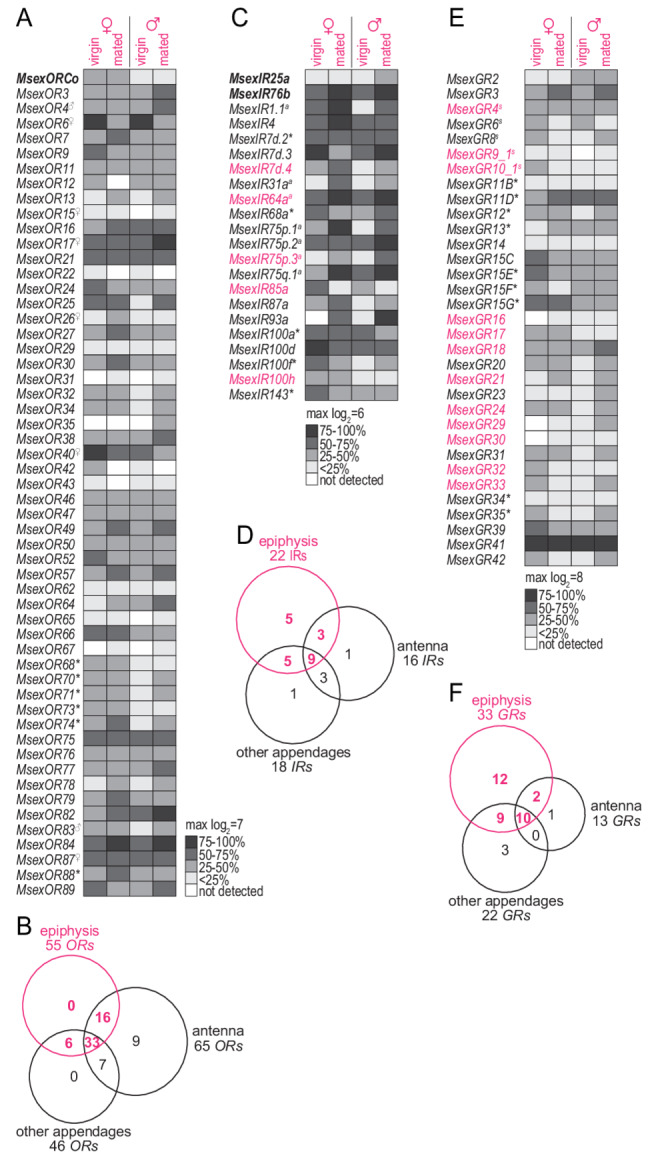
Expression of chemosensory receptor genes in the epiphysis of *M. sexta*. **A**, **C**, **E** Grayscale heatmaps showing the expression of *ORs* (A), *IRs* (C), and *GRs* (E) in the epiphysis of females (virgin/mated) and males (virgin/mated). Cells illustrate the normalized log_2_ of the geometric mean (*n* = 3 biological replicates) of normalized counts obtained from the NanoString assay; receptors are shown in the heatmap if they were considered to be expressed in at least one of the four groups (i.e., counts above background in at least 2 of the 3 biological replicates per group). *Bold gene names*, coreceptors; *light to dark shades of gray*, low to high expression levels; *white cells*, no detection of transcripts (i.e., counts at background level in all 3 biological replicates per group); ^♀^, female-biased expression in the antenna; ^♂^, male-biased expression in the antenna; *Not expressed in the antenna; *gene names in pink*, expressed only in the epiphysis; ^*a*^, putative acid-sensing *IR*; ^*s*^, putative sugar GR. **B**, **D**, **F** Venn diagrams showing the total number of *ORs* (B), *IRs* (D), and *GRs* (F) expressed in the epiphysis, antenna, and other appendages (mouthparts, legs, wings, ovipositor) and the number of receptors shared or unique among these 3 groups. Data for the antenna and other appendages are from (Tom et al. 2022)

The presence of functional olfactory sensory neurons in sensilla on the epiphysis requires the expression of olfactory receptors, which mainly belong to two chemosensory gene families: *ORs*, which in insects usually detect odorants from different chemical classes, and *IRs*, which detect mainly acids, aldehydes, and amines (Silbering et al. 2011). In addition, *GRs* in flies, mosquitoes, and moths have been shown to mediate sensing of CO_2_ and other odorants (Kumar et al. 2020; Ning et al. 2016; Tauxe et al. 2013). We used a custom code set targeting receptor genes from all three gene families (Tom et al. 2022; Zhang et al. 2022) and the Nanostring technology to measure receptor expression in dissected epiphyses of male and female moths, both before and after mating (Fig. 3). We detected the expression of a total of 110 chemosensory receptors, none of which showed sex-biased expression (*p* ≥ 0.2, DESeq2 analysis). However, some of the receptors were differentially expressed depending on the mating status of the moth (Table 2).

In detail, we found 55 *ORs* in the epiphysis, including the obligate co-receptor *ORCo* (Fig. 3A). The expression of *ORCo* in tissues of males and females was furthermore confirmed by RT-PCR (Fig. S3A). Two of the *ORs* expressed in the epiphysis (*MsexOR4*, *83*) belong to the male pheromone receptor clade (Bastin-Heline et al. 2019; Koenig et al. 2015). Furthermore, six of the eight female-biased antennal *ORs* (Tom et al. 2022) were detected, of which three are among the most highly expressed *ORs* in the epiphysis (*MsexOR6*, *17*, and *40*). Only one epiphysis *OR* (*MsexOR6*) showed a mating status-dependent expression, i.e., was less expressed in mated males than in virgin ones (Table 2). When compared to expression data from other tissues (Tom et al. 2022), the epiphysis did not express any unique *ORs* (Fig. 3B), but it did express *ORs* that are not expressed in the antenna but in other appendages such as the proboscis, labial palps, legs, wings, and ovipositor (Fig. 3A, *ORs* with asterisks). These six non-antennal *ORs* may be involved in the close-range evaluation of plant volatiles, an idea supported by the fact that four of these *ORs* (*MsexOR68*, *70*, *71*, *88*) have been previously described to be expressed in larval antennae and mouthparts (Koenig et al. 2015).

**Table 2:** Differential expression analysis of chemosensory receptors in the epiphysis

Receptor family	Receptor (sex)	Log_2_(fold change)	Adjusted *p*-value (DESeq2 analysis)*
ORs	*MsexOR6* (♂)	4.3	*p* = 0.003, virgin > mated
IRs	*MsexIR75q.1* (♀) *MsexIR85a* (♀) *MsexIR93a* (♂)	2.2 5.2 3.0	*p* = 0.015, mated > virgin *p* = 0.004, virgin > mated *p* = 0.019, mated > virgin

*Receptors are listed if log_2_(fold change) > 2.0 and *p* < 0.025 (adjusted significance level for multiple comparisons)

From the *IR* gene family, the expression of 22 receptors was detected in the epiphysis (Fig. 3C). The presence of the co-receptors *IR25a* and *76b*, which was confirmed by RT-PCR (Fig. S3B), indicates that the epiphysis is able to smell amines (Vulpe and Menuz 2021). In addition, seven *IRs* with putative acid tuning were found to be expressed in the epiphysis (Fig. 3C, *IRs* with ^a^), although the expression of *IR8a*, the co-receptor for acid-sensing *IRs* (Silbering et al. 2011) was not detected using the NanoString technique. However, we could show the presence of *IR8a* transcripts in the male epiphysis using the more sensitive RT-PCR (Fig. S3B). Expression levels of three of the *IRs* were dependent on mating status (Table 2). Five epiphysis *IRs* were not previously found in the antenna or other tissues (Tom et al. 2022) (Fig. 3C, *IRs* in pink), whereas of the *IRs* expressed in the antenna and other appendages with putative chemosensory function, only a single exclusive *IR* was found in those organs (Fig. 3D).

Additionally, we detected a total of 33 *GRs* (Fig. 3E), which is a considerably higher number than that found previously in the antennae and other appendages, including the mouthparts (Tom et al. 2022) (Fig. 3F). None of the epiphysis *GRs* changed expression upon mating (*p* ≥ 0.7, DESeq2 analysis). Two of the three members of the CO_2_ receptor gene subfamily (*MsexGR2* and *MsexGR3*) were expressed in the epiphysis. Next to three potential sugar receptors (Fig. 3E, *GRs* with ^s^), most of the other *GRs*, such as *MsexGR41*(the gene with the highest expression in the epiphysis (Fig. 3E) and other tissues (Tom et al. 2022), belong to the bitter receptor group. Many of these bitter receptors, as well as two sugar receptors (*MsexGR9_1* and *MsexGR10_1)*, were expressed exclusively in the epiphysis compared to the antennae and other appendages (Fig. 3F, *GRs* in pink).

Taken together, the expression patterns of chemosensory genes show that the epiphysis has a rich and partly exclusive chemosensory potential.

### Does removal of the epiphysis affect mating behavior?

**Fig. 4:**
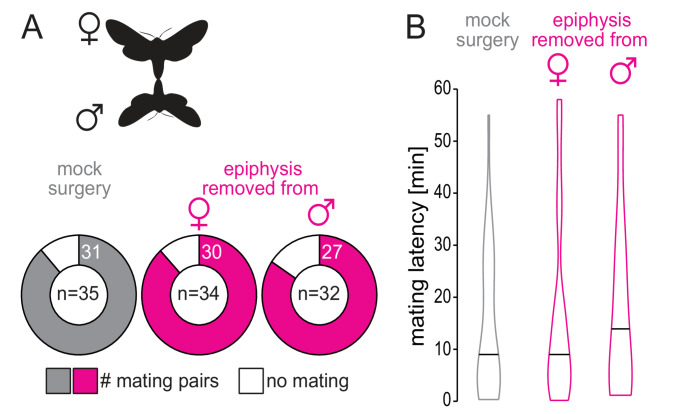
Effect of the removal of the epiphysis on mating behavior. A male and a female moth were free to mate in a cage for 60 min. The epiphyses on both forelegs were removed either in the female or the male, and the opposite sex underwent mock surgery (i.e., the animals were handled in the same way and for the same amount of time, but the epiphyses were not removed). In control experiments, both sexes underwent mock surgery. **A** *Donut plot*, proportion of mating pairs (number in donut area depicts absolute number), sample size is given in the center of each donut plot. *Gray*, mock surgery; *pink*, epiphysis removed from either female or male; pairwise Fisher’s exact tests. **B** Time until mating started. *Violin plot*, distribution of data, *horizontal line*, median; Kruskal-Wallis test

The response of the male epiphysis to the female pheromone gland and the expression of pheromone receptors suggest that the epiphysis may play a role in courtship. Therefore, surgical removal of the epiphysis could result in impaired courtship behavior. However, 84%−89% of the tested couples mated within the first hour, regardless of whether the male or the female underwent epiphysectomy or if both animals underwent mock surgery (Fig. 4A). We also analyzed mating latency but again saw no difference between the control group and the two experimental groups (Fig. 4B), suggesting that a lack of sensory input from the epiphysis does not delay courtship.

### Does removal of the epiphysis affect foraging and oviposition behavior?

**Fig. 5:**
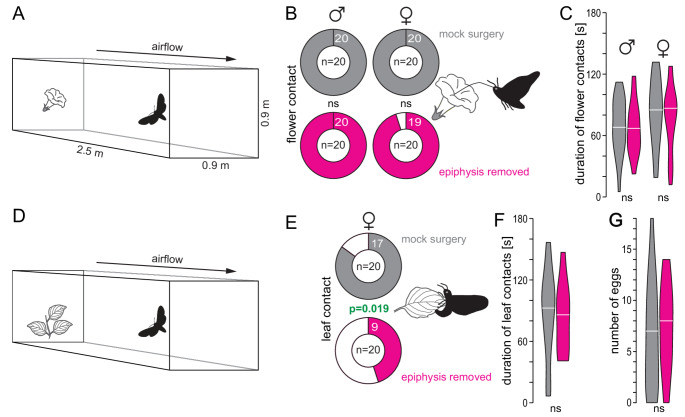
Effect of the removal of the epiphysis on foraging and oviposition behavior. **A** Schematic of the wind tunnel with a *D. wrightii* flower at the upwind end; male or female moths were released individually, and their behavior was recorded for 3 min. **B** Number of moths that made contact with the flower. *Gray*, mock surgery, *pink*, epiphysis removed; Fisher’s exact test. **C** Summed duration of flower contacts; Mann-Whitney *U* test. **D** Schematic of the wind tunnel with a potted *D. wrightii* plant with three leaves at the upwind end; mated female moths were released individually, their behavior was recorded for 3 min, and the number of eggs laid during this time was counted. **E** Number of mated females that made contact with the leaves. *Gray*, mock surgery, *pink*, epiphysis removed; Fisher’s exact test. **F** Summed duration of leaf contacts; Mann-Whitney *U* test. **G** Number of eggs laid on the leaves; Mann-Whitney *U* test

Since several plant-released volatiles elicited a response from the epiphysis, we investigated the role of this organ in foraging and oviposition contexts by conducting wind tunnel experiments with epiphysectomized animals and a control group that underwent mock surgery. Individual starved moths were released at the downwind end of the wind tunnel and observed for 3 min after flight initiation. In foraging experiments (Fig. 5A), we recorded if the moth touched a *D. wrightii* flower positioned at the upwind end of the wind tunnel with its proboscis and analyzed the total contact duration with the flower. We found that, in both the control and epiphysectomized groups, 98–100% of the moths touched the flower (Fig. 5B), and the cumulative flower contact duration was similar in both groups (Fig. 5C). In oviposition experiments (Fig. 5D), we placed a non-flowering *D. wrightii* plant at the upwind end of the wind tunnel and tested it with individual mated females. We observed that 85% of females in the control group but only 45% of females in the epiphysectomized group contacted the host plant with their tarsi (*p* = 0.019, Fisher’s exact test, Fig. 5E). However, there was no difference in total contact time and number of eggs laid between the control and experimental groups of moths that contacted the leaf (Fig. 5F, G).

These results suggest that the epiphysis is not necessary for foraging behavior in *M. sexta*, but may play a supporting role for gravid females in locating *D. wrightii* plants. However, oviposition behavior once the moth has made contact with the host plant leaf was not dependent on the presence of the epiphysis.

## Discussion

Our study shows that the epiphysis on the foreleg tibia of *M. sexta* may be both a grooming organ and an olfactory organ. A first clue was the presence of sensilla with a porous cuticle, a prerequisite for the olfactory function of a sensillum. Because wall-pore sensilla were found only on the most proximal part of the epiphysis, near its insertion site on the tibia, they may have been previously overlooked in *M. sexta* (Kent and Griffin 1990) and other moth species (Marion-Poll et al. 1992). The number of wall-pore sensilla per epiphysis was about 150 in both sexes, suggesting that the olfactory capacity of the epiphysis could be higher than that of other accessory olfactory appendages, such as the proboscis (1 olfactory sensillum) (Haverkamp et al. 2016) and the ovipositor (4–9 olfactory sensilla) (Klinner et al. 2016).

Electrophysiological recordings from isolated epiphyses revealed a broad receptive range, with active ligands belonging to several chemical classes. Additionally, the male epiphysis responded to an extract from the female pheromone gland. These findings are consistent with the observed expression of the obligate co-receptor *ORCo* and 54 tuning *ORs* in the epiphysis. One receptor detected in the epiphysis was *MsexOR4*, a pheromone receptor with the highest expression level of all *ORs* in the male antenna (Koenig et al. 2015). Furthermore, *MsexOR4* expression was not detected in other previously studied appendages (Tom et al. 2022). Therefore, we speculated that the epiphysis of *M. sexta* may play a role in the sequence of precopulatory behaviors, as has been reported for the spongy moth (Odell et al. 1982). Interestingly, other arthropods, such as orb-weaving spiders have also been shown to possess wall-pore sensilla on their legs that house pheromone-sensing neurons (Talukder et al. 2025). However, when we surgically removed the epiphyses from male (or female) *M. sexta*, mating latency and success did not differ from control pairs. Nevertheless, the unusual expression of *MsexOR4* in the epiphysis, as well its response to the pheromone gland, suggest that the epiphysis may contribute to pheromone communication in other ways. For example, it could facilitate sexual isolation from sympatric hawkmoth species (Alarcon et al. 2008), which may use different pheromone blends.

We demonstrated that the epiphysis may play a role in female-specific behaviors, such as evaluating host plants at close range. This is evident because the headspace of host plant leaves activated the female epiphysis, and a lower proportion of epiphysectomized females than controls reached and contacted a host plant.

The strong activation of the epiphysis by acids also suggests that this foreleg tissue may be involved in oviposition behaviors. Acids released from the feces of larval conspecifics have been shown to repel ovipositing female moths (Schuh et al. 2024; Zhang et al. 2019). Accordingly, we identified several acid-sensing *IRs* in the epiphysis. One of these is *MsexIR75p.1*, which is an ortholog of the *IR* responsible for detecting repellent acids in the noctuid moth *Agrotis segetum* (Hou et al. 2022). *MsexIR75p.1* was expressed at higher levels in the epiphysis of mated female *M. sexta*, supporting the idea that this *IR* plays a role in oviposition decision-making. Olfactory responses to acids are typically mediated by acid-sensing *IRs* together with the co-receptor *IR8a* (Silbering et al. 2011). However, since we only demonstrated *IR8a* expression in the male epiphysis, our data suggest that the acid response in the epiphysis may be *IR8a*-independent.

Furthermore, we could show a robust electrophysiological response of the epiphysis to pyrrolidine, an amine that exhibited no behavioral effects in wind tunnel experiments (Bisch-Knaden et al. 2018). However, this odor may signal important host plants to *M. sexta* because pyrrolidine alkaloids have been identified in *Datura* (Cinelli and Jones 2021) and other Solanaceae plants (Pomilio et al. 2008). Given the epiphysis’s clear response to pyrrolidine, we expected to find orthologs of the pyrrolidine-sensing *IRs* identified in *D. melanogaster* (Silbering et al. 2011). The expression of these orthologs (*MsexIR41a* and *75d*, (Koenig et al. 2015) has been demonstrated in the pyrrolidine-sensing antennae and ovipositors of *M. sexta* (Bisch-Knaden et al. 2018; Klinner et al. 2016; Tom et al. 2022). However, *MsexIR41a* and *75d* could not be detected in the epiphysis. Therefore, pyrrolidine may be a ligand of *IRs* without an ortholog in *D. melanogaster*. One example is the *IR7* clade, which is specific to Lepidoptera and has not yet been functionally characterized. Alternatively, pyrrolidine may be detected by other chemosensory receptors expressed in the epiphysis.

Typical gustatory sensilla with an apical pore were absent from the epiphysis, which is a tissue that usually does not come into contact with the surface. Nevertheless, we detected the expression of numerous *GRs*. The expression of chemosensory receptor genes has been found not only in tissues at the exterior of the insect body that have access to or contact with the environment, but also in internal tissues, such as the brain and reproductive organs. In these tissues, the receptors sense nutrients in the hemolymph (Miyamoto et al. 2012) or are involved in fertility regulation (David et al. 2023). Therefore, the *GRs* detected in the epiphysis may play an internal chemosensory role. Additionally, some epiphysis *GRs* may have an olfactory function and be expressed by sensory neurons in wall-pore sensilla, either alone or coexpressed in neurons that express *ORs* or *IRs* (Herre et al. 2022). One example of *GRs* with an olfactory function is the conserved CO_2_-sensing receptor clade found in several insects. This clade consists of two *GRs* in flies and three GRs in mosquitoes and moths (Jones et al. 2007; Lu et al. 2007; Ning et al. 2016). In both moths and mosquitoes, only two *GRs* in the clade are required for CO_2_ sensing; the third receptor has a modulatory or unknown function. *GR1* is required together with *GR3* for CO_2_ binding in other moth species (Ning et al. 2016; Zhang et al. 2024). Therefore, the absence of *GR1* expression in the epiphysis of *M. sexta* suggests that this tissue is not involved in CO_2_ sensing. Instead, *MsexGR2* and *3* may detect additional odorants, as demonstrated in vinegar flies and mosquitoes (Kumar et al. 2020; MacWilliam et al. 2018; Tauxe et al. 2013). Interestingly, the odorants that activate *GR2* and *GR3* in those insects include acids and amines. Thus, the acid and amine responses of the epiphyses of *M. sexta* may be mediated by these two gustatory receptors.

Taken together, our findings show that the epiphysis on the forelegs of *M. sexta* is an olfactory organ that was previously unknown and that it may be a complementary part of the moth’s chemosensory system. Because an epiphysis is a characteristic feature of nearly all Lepidoptera, it appears reasonable to assume that this tissue serves a similar function in other moths and butterflies.

## Supplementary Information

Below is the link to the electronic supplementary material.

Supplementary Material 1

## Data Availability

No datasets were generated or analysed during the current study.
