# Supplementary material for: Hawkmoths can smell with grooming organs on their legs
Source: J Comp Physiol A Neuroethol Sens Neural Behav Physiol. 2025 Oct 25;212(2):197–209. doi: 10.1007/s00359-025-01769-y (PMC13086689; doi:10.1007/s00359-025-01769-y)
Supplement: Supplementary file 1 — Supplementary Material 1 [file 359_2025_1769_MOESM1_ESM.pdf]

Table S1. List of odorants.

odorant	CAS number
(+)-linalool	126-90-9
2-methylbutylaldoxime (e/z)	49805-55-2 / 49805-56-3
2-phenylethanol	60-12-8
3-methylbutylaldoxime (e/z)	5775-74-6 / 5780-40-5
acetic acid	64-19-7
acetophenone	98-86-2
benzyl acetone	2550-26-7
benzyl alcohol	100-51-6
butyl butyrate	109-21-7
diethyltoluamide (DEET)	134-62-3
E2-hexenal	6728-26-3
ethyl hexanoate	123-66-0
γ-hexalactone	695-06-7
heptanoic acid	111-14-8
hexanoic acid	142-62-1
isovaleric acid	503-74-2
methyl benzoate	93-58-3
methyl hexanoate	106-70-7
octanoic acid	124-07-2
p-cresol	106-44-5
phenyl acetaldehyde	122-78-1
pyrrolidine	123-75-1
Z3-hexenol	928-96-1
Z3-hexenyl acetate	3681-71-8
Z3-hexenyl propionate	33467-74-2

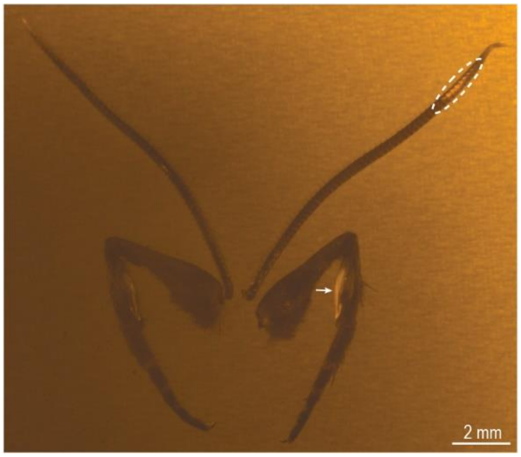

Figure S1. Antennal grooming with the epiphysis.

Fluorescent powder was applied to the epiphysis on the right foreleg of a male moth. After 24 h, the powder was present on the right antenna.

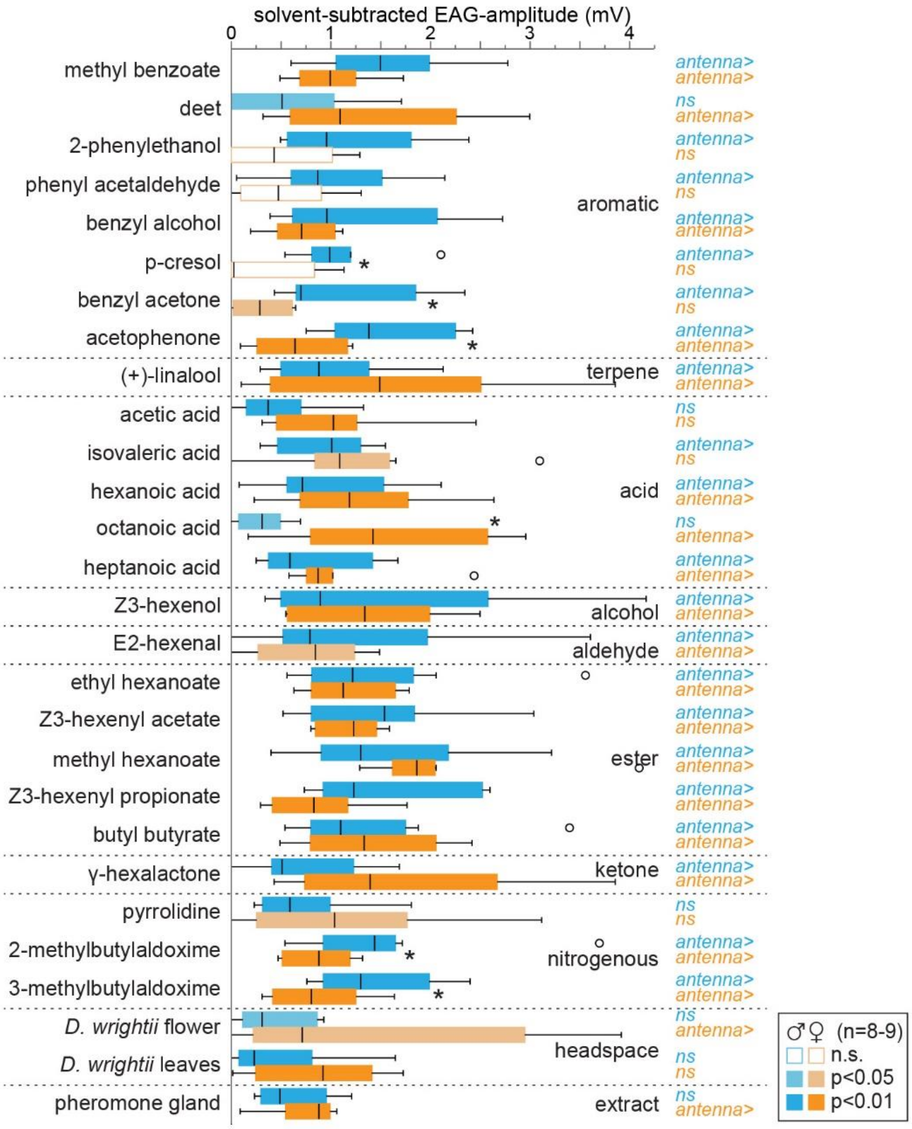

Figure S2. Electrophysiological recordings from the antenna (EAG).

EAG responses of male (blue) and female (orange) *M. sexta* epiphyses to 25 odorants from eight chemical classes (10 µl, diluted 1:100), headspace (10 µl) of *D. wrightii* (flower and leaves separately), and extract (10 µl) from female pheromone glands. *Boxplots*, median, interquartile range and range; *circles*, outliers; *filled boxes*, data different from zero (p<0.05 (light blue/orange) or p<0.01 (dark blue/orange), Wilcoxon signed rank test); *asterisk*, difference between the sexes (Mann-Whitney U test); *right column*, comparison between recordings from antennae and epiphyses (see Fig. 2); *antenna>*, responses from antennae stronger than from epiphyses (p<0.05); *ns*, no difference between responses from antennae and epiphyses (p>0.05, Mann-Whitney U test); the Bonferroni-Holmes method was used to adjust the significance level for multiple comparisons.

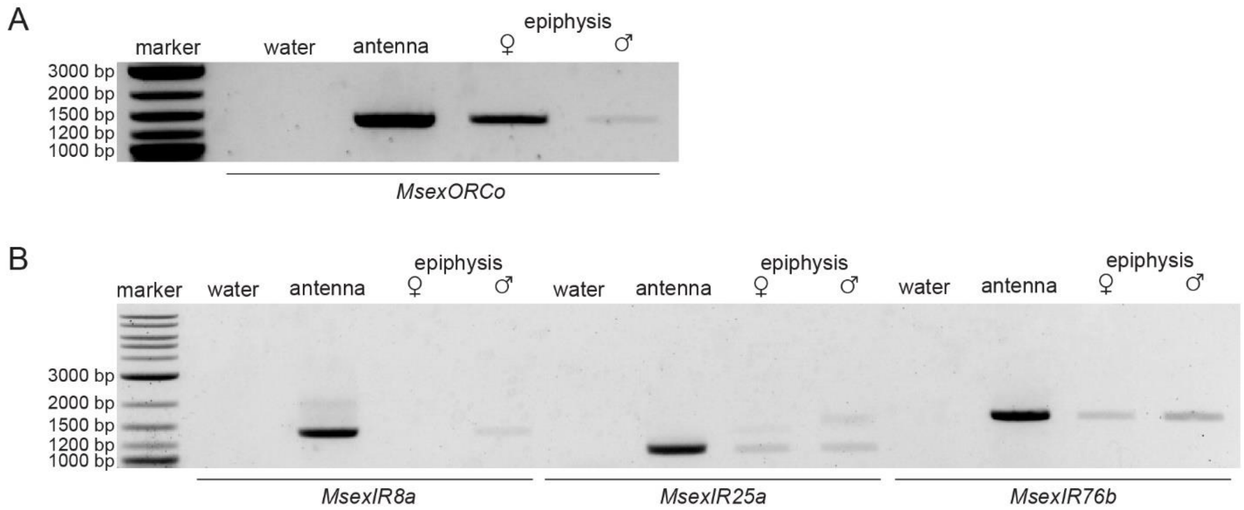

Figure S3. Reverse transcriptase-PCR amplifications of the OR co-receptor *ORCo* (A) and the IR-co-receptors *IR8a*, *IR25a*, and *IR76b* (B) in the epiphyses of females and males.

RNA extracted from the antenna served as the positive control, and water as the negative control.
